# *catena*-Poly[tris(2,4,6-trimethyl­anilinium) [(tetrachloridocadmium)-μ-chlorido]]

**DOI:** 10.1107/S1600536811028650

**Published:** 2011-07-23

**Authors:** Tao Rong

**Affiliations:** a Ordered Matter Science Research Center, Southeast University, Nanjing 210096, People’s Republic of China

## Abstract

The asymmetric unit of the title compound, {(C_9_H_14_N)_3_[CdCl_5_]}_*n*_, comprises three 2,4,6-trimethyl­aniline dications and one half of the [Cd_2_Cl_10_]^6−^ anion. The Cd atoms are each coordinated by six Cl atoms, with octa­hedra linked by bridging, apical Cl atoms, forming linear chains running parallel to the *a* axis. The trimethylanilinium cations form stacks between the chains of CdCl_6_ octa­hedra.

## Related literature

The title compound was studied as part of our work to obtain potential ferroelectric phase-change materials. For general background to ferroelectric metal-organic frameworks, see: Fu *et al.* (2009 ▶); Ye *et al.* (2006 ▶); Zhang *et al.* (2008 ▶, 2010 ▶).
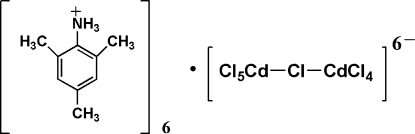


## Experimental

### 

#### Crystal data

(C_9_H_14_N)_3_[CdCl_5_]
*M* *_r_* = 698.29
Orthorhombic, 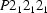

*a* = 10.729 (2) Å
*b* = 16.430 (3) Å
*c* = 17.996 (4) Å
*V* = 3172.2 (11) Å^3^
*Z* = 4
Mo *K*α radiation
μ = 1.13 mm^−1^
*T* = 293 K
0.20 × 0.20 × 0.20 mm

#### Data collection

Rigaku SCXmini diffractometer
Absorption correction: multi-scan (*CrystalClear*; Rigaku, 2005 ▶) *T*_min_ = 0.798, *T*_max_ = 0.798
33173 measured reflections
7271 independent reflections
6752 reflections with *I* > 2σ(*I*)
*R*_int_ = 0.046

#### Refinement

*R*[*F* ^2^ > 2σ(*F* ^2^)] = 0.033
*wR*(*F* ^2^) = 0.068
*S* = 1.07
7271 reflections
337 parameters
H-atom parameters constrained
Δρ_max_ = 0.30 e Å^−3^
Δρ_min_ = −0.53 e Å^−3^

### 

Data collection: *CrystalClear* (Rigaku, 2005 ▶); cell refinement: *CrystalClear*; data reduction: *CrystalClear*; program(s) used to solve structure: *SHELXS97* (Sheldrick, 2008 ▶); program(s) used to refine structure: *SHELXL97* (Sheldrick, 2008 ▶); molecular graphics: *DIAMOND* (Brandenburg & Putz, 2005 ▶); software used to prepare material for publication: *SHELXL97*.

## Supplementary Material

Crystal structure: contains datablock(s) I, global. DOI: 10.1107/S1600536811028650/jh2312sup1.cif

Structure factors: contains datablock(s) I. DOI: 10.1107/S1600536811028650/jh2312Isup2.hkl

Additional supplementary materials:  crystallographic information; 3D view; checkCIF report

## Figures and Tables

**Table 1:** Hydrogen-bond geometry (Å, °)

*D*—H⋯*A*	*D*—H	H⋯*A*	*D*⋯*A*	*D*—H⋯*A*
N2—H2*C*⋯Cl3	0.89	2.26	3.129 (3)	166
N3—H3*B*⋯Cl3	0.89	2.70	3.283 (3)	124
N3—H3*B*⋯Cl5	0.89	2.62	3.158 (3)	119
N2—H2*A*⋯Cl6^i^	0.89	2.40	3.250 (3)	160
N3—H3*A*⋯Cl2^ii^	0.89	2.41	3.264 (3)	160
N1—H1*A*⋯Cl4^iii^	0.89	2.43	3.278 (3)	160
N1—H1*B*⋯Cl3^iv^	0.89	2.61	3.285 (3)	134
N1—H1*C*⋯Cl2^iv^	0.89	2.43	3.306 (3)	169

Symmetry codes: (i) 
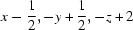
; (ii) 
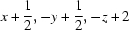
; (iii) 
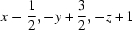
; (iv) 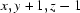.

**Fig. 1.**
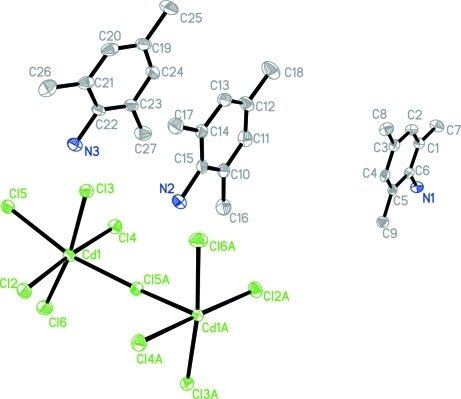
The molecular structure of the title compound, with the atomic numbering scheme. Displacement ellipsoids are drawn at the 30% probability level.[The suffix A denotes the symmetry code: -1/2 + x 0.5 - y 2 - z]

**Fig. 2.**
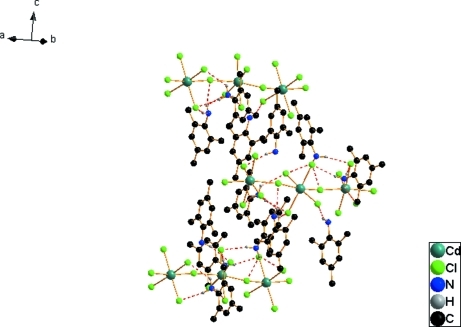
A view of the packing of the title compound, stacking along the a axis. Dashed lines indicate hydrogen bonds.

## supplementary crystallographic information

### Comment

The study of ferroelectric materials has received much attention. Some materials have predominantly dielectric-ferroelectric performance. The title compound was studied as part of our work to obtain potential ferroelectric phase-change materials Fu *et al.*(2009); Ye *et al.* (2006); Zhang *et al.* (2008, 2010).

As one part of our continuing studies on dielectric-ferroelectric materials, we synthesized the title compound (C_9_H_14_N)_3_.CdCl_5._ Unfortunately, the study carried out on the title compound indicated that the permittivity is temperature-independent, suggesting that there may be no dielectric disuniformity between 80 K to 350 K.

Theasymmetric unit of the title compound contains three [C_9_H_47_N]^+^ basic ion and half of the [Cd_2_Cl_10_]^6-^ complex ionwhich is situated on an inversion centre. The intermolecular hydrogen bonds (N1—H···Cl2, N1—H···Cl3, N1—H···Cl4, N2—H···Cl3, N2—H···Cl6, N3—H···Cl2, N3—H···Cl3 and N3—H···Cl5 link the molecules into a one-dimensional linear structure and stabilize the structure.

### Experimental

A solution of chlorhydric acid (10 mmol) was added to a solution of half equimolar amount of 2,4,6-Trimethylaniline inethanol (20 mL), then cadmium chloride(5 mmol) in water (10 mL) was added.Crystals suitable for structure determination were grown by slow evaporation of the mixture at room temperature.

### Refinement

Positional parameters of all the H atoms bonded to C atoms were calculated geometrically and were allowed to ride on the C atoms to which they are bonded, with *U*_iso_(H) = 1.2Ueq(C) and *U*_iso_(H) = 1.5Ueq(C) for the methyl group. The other H bonded to N atoms were calculated geometrically and were allowed to ride on the N atoms with *U*_iso_(H) = 1.2Ueq(N).

### Crystal data

**Table:**

(C_9_H_14_N)_3_[CdCl_5_]	*F*(000) = 1432
*M**_r_* = 698.29	*D*_x_ = 1.462 Mg m^−^^3^
Orthorhombic, *P*2_1_2_1_2_1_	Mo *K*α radiation, λ = 0.71073 Å
Hall symbol: P 2ac 2ab	Cell parameters from 7271 reflections
*a* = 10.729 (2) Å	θ = 3.1–27.5°
*b* = 16.430 (3) Å	µ = 1.13 mm^−^^1^
*c* = 17.996 (4) Å	*T* = 293 K
*V* = 3172.2 (11) Å^3^	Prism, colourless
*Z* = 4	0.20 × 0.20 × 0.20 mm

### Data collection

**Table:**

Rigaku SCXmini diffractometer	7271 independent reflections
Radiation source: fine-focus sealed tube	6752 reflections with *I* > 2σ(*I*)
graphite	*R*_int_ = 0.046
Detector resolution: 13.6612 pixels mm^-1^	θ_max_ = 27.5°, θ_min_ = 3.1°
CCD_Profile_fitting scans	*h* = −13→13
Absorption correction: multi-scan (*CrystalClear*; Rigaku, 2005)	*k* = −21→21
*T*_min_ = 0.798, *T*_max_ = 0.798	*l* = −23→23
33173 measured reflections	

### Refinement

**Table:**

Refinement on *F*^2^	Primary atom site location: structure-invariant direct methods
Least-squares matrix: full	Secondary atom site location: difference Fourier map
*R*[*F*^2^ > 2σ(*F*^2^)] = 0.033	Hydrogen site location: inferred from neighbouring sites
*wR*(*F*^2^) = 0.068	H-atom parameters constrained
*S* = 1.07	*w* = 1/[σ^2^(*F*_o_^2^) + (0.0235*P*)^2^ + 1.6716*P*] where *P* = (*F*_o_^2^ + 2*F*_c_^2^)/3
7271 reflections	(Δ/σ)_max_ = 0.006
337 parameters	Δρ_max_ = 0.30 e Å^−^^3^
0 restraints	Δρ_min_ = −0.53 e Å^−^^3^

### Special details

**Table:**

Geometry. All e.s.d.'s (except the e.s.d. in the dihedral angle between two l.s. planes) are estimated using the full covariance matrix. The cell e.s.d.'s are taken into account individually in the estimation of e.s.d.'s in distances, angles and torsion angles; correlations between e.s.d.'s in cell parameters are only used when they are defined by crystal symmetry. An approximate (isotropic) treatment of cell e.s.d.'s is used for estimating e.s.d.'s involving l.s. planes.
Refinement. Refinement of *F*^2^ against ALL reflections. The weighted *R*-factor *wR* and goodness of fit *S* are based on *F*^2^, conventional *R*-factors *R* are based on *F*, with *F* set to zero for negative *F*^2^. The threshold expression of *F*^2^ > σ(*F*^2^) is used only for calculating *R*-factors(gt) *etc*. and is not relevant to the choice of reflections for refinement. *R*-factors based on *F*^2^ are statistically about twice as large as those based on *F*, and *R*- factors based on ALL data will be even larger.

### Fractional atomic coordinates and isotropic or equivalent isotropic displacement parameters (Å^2^)

**Table:**

	*x*	*y*	*z*	*U*_iso_*/*U*_eq_	
Cd1	0.612594 (19)	0.264994 (14)	1.010000 (11)	0.03061 (6)	
Cl2	0.57453 (8)	0.19719 (5)	1.13969 (4)	0.0422 (2)	
Cl3	0.59250 (7)	0.12522 (5)	0.93873 (4)	0.03403 (17)	
Cl4	0.63550 (8)	0.33959 (5)	0.88122 (5)	0.03973 (19)	
Cl5	0.86375 (6)	0.22240 (5)	1.00838 (4)	0.04127 (19)	
Cl6	0.67019 (9)	0.39688 (6)	1.07986 (6)	0.0501 (2)	
N2	0.3915 (3)	0.19586 (18)	0.83050 (14)	0.0433 (7)	
H2A	0.3185	0.1765	0.8460	0.065*	
H2B	0.3952	0.2491	0.8394	0.065*	
H2C	0.4529	0.1708	0.8546	0.065*	
C15	0.4047 (3)	0.1812 (2)	0.74953 (17)	0.0328 (7)	
N3	0.8225 (3)	0.19469 (18)	0.83647 (15)	0.0407 (7)	
H3A	0.8995	0.2148	0.8355	0.061*	
H3B	0.8135	0.1631	0.8763	0.061*	
H3C	0.7678	0.2354	0.8385	0.061*	
C6	0.3959 (3)	0.97807 (18)	0.08418 (17)	0.0336 (7)	
C10	0.3967 (3)	0.24689 (18)	0.70168 (17)	0.0360 (7)	
C5	0.3917 (3)	0.96051 (19)	0.15963 (16)	0.0346 (7)	
N1	0.3849 (3)	1.06423 (16)	0.06078 (16)	0.0449 (7)	
H1A	0.3075	1.0816	0.0690	0.067*	
H1B	0.4024	1.0684	0.0126	0.067*	
H1C	0.4382	1.0945	0.0867	0.067*	
C1	0.4063 (3)	0.9183 (2)	0.03014 (17)	0.0360 (7)	
C16	0.3799 (4)	0.3324 (2)	0.7294 (2)	0.0506 (9)	
H16A	0.3039	0.3360	0.7574	0.076*	
H16B	0.3761	0.3691	0.6879	0.076*	
H16C	0.4490	0.3469	0.7607	0.076*	
C23	0.7664 (3)	0.1862 (2)	0.7048 (2)	0.0393 (8)	
C27	0.7503 (4)	0.2771 (3)	0.7006 (2)	0.0553 (11)	
H27A	0.6782	0.2929	0.7286	0.083*	
H27B	0.7397	0.2931	0.6496	0.083*	
H27C	0.8228	0.3034	0.7207	0.083*	
C4	0.4004 (3)	0.8788 (2)	0.18055 (18)	0.0382 (7)	
H4	0.3989	0.8657	0.2308	0.046*	
C3	0.4112 (3)	0.8174 (2)	0.12931 (19)	0.0378 (8)	
C14	0.4213 (3)	0.1022 (2)	0.72525 (18)	0.0344 (7)	
C9	0.3765 (4)	1.0260 (2)	0.21723 (19)	0.0460 (9)	
H9A	0.4447	1.0637	0.2137	0.069*	
H9B	0.3757	1.0019	0.2658	0.069*	
H9C	0.2995	1.0543	0.2090	0.069*	
C11	0.4044 (3)	0.2298 (2)	0.62569 (18)	0.0445 (8)	
H11	0.4005	0.2726	0.5920	0.053*	
C7	0.4097 (5)	0.9373 (2)	−0.05204 (19)	0.0522 (10)	
H7A	0.4154	0.8875	−0.0798	0.078*	
H7B	0.4809	0.9707	−0.0627	0.078*	
H7C	0.3351	0.9658	−0.0658	0.078*	
C2	0.4145 (3)	0.8384 (2)	0.05417 (19)	0.0414 (8)	
H2	0.4225	0.7972	0.0190	0.050*	
C21	0.8163 (3)	0.0623 (2)	0.7733 (2)	0.0409 (9)	
C22	0.8005 (3)	0.1463 (2)	0.76894 (19)	0.0344 (7)	
C19	0.7601 (4)	0.0551 (3)	0.6430 (2)	0.0533 (11)	
C12	0.4174 (3)	0.1513 (2)	0.59900 (18)	0.0447 (9)	
C13	0.4265 (3)	0.0884 (2)	0.64915 (19)	0.0406 (8)	
H13	0.4363	0.0355	0.6317	0.049*	
C20	0.7946 (3)	0.0186 (2)	0.7084 (2)	0.0497 (10)	
H20	0.8038	−0.0376	0.7093	0.060*	
C26	0.8547 (4)	0.0203 (3)	0.8438 (2)	0.0601 (12)	
H26A	0.7911	0.0275	0.8808	0.090*	
H26B	0.9315	0.0432	0.8614	0.090*	
H26C	0.8661	−0.0368	0.8344	0.090*	
C17	0.4321 (4)	0.0315 (2)	0.7783 (2)	0.0498 (10)	
H17A	0.5117	0.0334	0.8026	0.075*	
H17B	0.4246	−0.0186	0.7512	0.075*	
H17C	0.3670	0.0348	0.8147	0.075*	
C24	0.7476 (3)	0.1390 (3)	0.6415 (2)	0.0498 (10)	
H24	0.7262	0.1645	0.5971	0.060*	
C8	0.4193 (4)	0.7297 (2)	0.1520 (2)	0.0523 (9)	
H8A	0.4091	0.7254	0.2048	0.078*	
H8B	0.4992	0.7082	0.1380	0.078*	
H8C	0.3548	0.6993	0.1276	0.078*	
C25	0.7373 (5)	0.0056 (4)	0.5723 (3)	0.0867 (18)	
H25A	0.7478	−0.0513	0.5829	0.130*	
H25B	0.7957	0.0218	0.5347	0.130*	
H25C	0.6540	0.0151	0.5549	0.130*	
C18	0.4226 (4)	0.1344 (3)	0.5161 (2)	0.0678 (12)	
H18A	0.5070	0.1392	0.4990	0.102*	
H18B	0.3711	0.1729	0.4903	0.102*	
H18C	0.3928	0.0803	0.5065	0.102*	

### Atomic displacement parameters (Å^2^)

**Table:**

	*U*^11^	*U*^22^	*U*^33^	*U*^12^	*U*^13^	*U*^23^
Cd1	0.02973 (10)	0.03547 (11)	0.02664 (10)	−0.00434 (10)	0.00074 (9)	−0.00228 (9)
Cl2	0.0450 (5)	0.0514 (5)	0.0302 (4)	0.0034 (4)	0.0016 (3)	0.0044 (4)
Cl3	0.0329 (4)	0.0339 (4)	0.0354 (4)	−0.0027 (3)	0.0002 (3)	−0.0018 (3)
Cl4	0.0413 (5)	0.0397 (4)	0.0381 (4)	−0.0007 (4)	0.0021 (4)	0.0066 (3)
Cl5	0.0268 (3)	0.0618 (5)	0.0352 (4)	−0.0017 (3)	−0.0009 (3)	−0.0086 (4)
Cl6	0.0518 (5)	0.0427 (5)	0.0559 (6)	−0.0007 (4)	−0.0048 (4)	−0.0157 (4)
N2	0.0499 (18)	0.0493 (17)	0.0309 (14)	0.0036 (17)	−0.0053 (15)	−0.0059 (12)
C15	0.0273 (17)	0.0417 (18)	0.0293 (16)	−0.0013 (15)	−0.0032 (14)	−0.0004 (13)
N3	0.0399 (17)	0.0464 (17)	0.0359 (16)	−0.0066 (14)	0.0037 (13)	−0.0087 (13)
C6	0.0281 (16)	0.0309 (16)	0.0418 (18)	−0.0012 (15)	0.0027 (15)	0.0076 (13)
C10	0.0297 (15)	0.039 (2)	0.0395 (16)	−0.0038 (16)	−0.0022 (13)	0.0020 (13)
C5	0.0260 (15)	0.0423 (18)	0.0354 (17)	−0.0028 (16)	0.0003 (15)	0.0017 (13)
N1	0.0512 (17)	0.0363 (15)	0.0472 (16)	−0.0002 (16)	0.0082 (17)	0.0031 (12)
C1	0.0361 (18)	0.0374 (17)	0.0344 (17)	−0.0040 (15)	0.0038 (14)	0.0061 (13)
C16	0.057 (2)	0.039 (2)	0.056 (2)	−0.001 (2)	−0.008 (2)	0.0017 (16)
C23	0.0343 (19)	0.046 (2)	0.038 (2)	−0.0010 (16)	0.0051 (15)	−0.0101 (17)
C27	0.067 (3)	0.053 (3)	0.046 (2)	0.004 (2)	0.0077 (19)	0.000 (2)
C4	0.0294 (17)	0.0496 (19)	0.0357 (17)	0.0032 (17)	0.0004 (15)	0.0112 (14)
C3	0.0311 (18)	0.0413 (18)	0.0409 (18)	−0.0002 (15)	0.0032 (15)	0.0119 (15)
C14	0.0295 (17)	0.0386 (18)	0.0352 (17)	0.0010 (14)	−0.0016 (13)	−0.0002 (14)
C9	0.048 (2)	0.051 (2)	0.0391 (19)	0.003 (2)	0.0048 (18)	−0.0005 (16)
C11	0.0442 (19)	0.0531 (19)	0.0362 (17)	−0.006 (2)	0.0026 (15)	0.0109 (17)
C7	0.077 (3)	0.041 (2)	0.039 (2)	−0.002 (2)	0.004 (2)	0.0055 (15)
C2	0.049 (2)	0.0358 (18)	0.0394 (19)	0.0017 (16)	0.0068 (16)	0.0021 (14)
C21	0.0284 (18)	0.041 (2)	0.053 (2)	−0.0032 (16)	0.0099 (16)	−0.0100 (17)
C22	0.0280 (17)	0.0413 (19)	0.0338 (18)	−0.0056 (15)	0.0045 (13)	−0.0151 (15)
C19	0.040 (2)	0.063 (3)	0.057 (3)	0.0045 (19)	0.0001 (18)	−0.031 (2)
C12	0.040 (2)	0.064 (2)	0.0304 (18)	−0.0109 (18)	0.0049 (14)	−0.0071 (16)
C13	0.0375 (19)	0.044 (2)	0.040 (2)	−0.0009 (16)	0.0019 (15)	−0.0092 (16)
C20	0.036 (2)	0.039 (2)	0.075 (3)	−0.0036 (17)	0.0062 (19)	−0.0208 (19)
C26	0.060 (3)	0.049 (2)	0.071 (3)	0.009 (2)	0.005 (2)	0.005 (2)
C17	0.060 (3)	0.044 (2)	0.046 (2)	0.006 (2)	−0.0082 (18)	−0.0009 (17)
C24	0.042 (2)	0.071 (3)	0.037 (2)	0.0054 (19)	−0.0033 (16)	−0.016 (2)
C8	0.060 (2)	0.0436 (19)	0.053 (2)	0.009 (2)	0.0059 (17)	0.0121 (19)
C25	0.079 (4)	0.101 (4)	0.080 (4)	0.014 (3)	−0.017 (3)	−0.060 (3)
C18	0.071 (3)	0.096 (3)	0.036 (2)	−0.015 (3)	0.008 (2)	−0.008 (2)

### Geometric parameters (Å, °)

**Table:**

Cd1—Cl6	2.5803 (10)	C3—C2	1.396 (4)
Cd1—Cl2	2.6182 (9)	C3—C8	1.500 (5)
Cd1—Cl4	2.6331 (10)	C14—C13	1.389 (5)
Cd1—Cl3	2.6393 (9)	C14—C17	1.508 (5)
Cd1—Cl5^i^	2.6981 (9)	C9—H9A	0.9600
Cd1—Cl5	2.7841 (9)	C9—H9B	0.9600
Cl5—Cd1^ii^	2.6981 (9)	C9—H9C	0.9600
N2—C15	1.484 (4)	C11—C12	1.383 (5)
N2—H2A	0.8900	C11—H11	0.9300
N2—H2B	0.8900	C7—H7A	0.9600
N2—H2C	0.8900	C7—H7B	0.9600
C15—C14	1.380 (5)	C7—H7C	0.9600
C15—C10	1.384 (4)	C2—H2	0.9300
N3—C22	1.471 (4)	C21—C20	1.391 (5)
N3—H3A	0.8900	C21—C22	1.393 (5)
N3—H3B	0.8900	C21—C26	1.503 (5)
N3—H3C	0.8900	C19—C20	1.370 (6)
C6—C1	1.387 (4)	C19—C24	1.386 (6)
C6—C5	1.389 (4)	C19—C25	1.530 (5)
C6—N1	1.482 (4)	C12—C13	1.375 (5)
C10—C11	1.398 (4)	C12—C18	1.519 (5)
C10—C16	1.502 (5)	C13—H13	0.9300
C5—C4	1.397 (4)	C20—H20	0.9300
C5—C9	1.503 (5)	C26—H26A	0.9600
N1—H1A	0.8900	C26—H26B	0.9600
N1—H1B	0.8900	C26—H26C	0.9600
N1—H1C	0.8900	C17—H17A	0.9600
C1—C2	1.385 (4)	C17—H17B	0.9600
C1—C7	1.512 (4)	C17—H17C	0.9600
C16—H16A	0.9600	C24—H24	0.9300
C16—H16B	0.9600	C8—H8A	0.9600
C16—H16C	0.9600	C8—H8B	0.9600
C23—C22	1.376 (5)	C8—H8C	0.9600
C23—C24	1.393 (5)	C25—H25A	0.9600
C23—C27	1.506 (5)	C25—H25B	0.9600
C27—H27A	0.9600	C25—H25C	0.9600
C27—H27B	0.9600	C18—H18A	0.9600
C27—H27C	0.9600	C18—H18B	0.9600
C4—C3	1.372 (5)	C18—H18C	0.9600
C4—H4	0.9300		
			
Cl6—Cd1—Cl2	87.73 (3)	C15—C14—C17	122.2 (3)
Cl6—Cd1—Cl4	90.89 (3)	C13—C14—C17	119.7 (3)
Cl2—Cd1—Cl4	175.69 (3)	C5—C9—H9A	109.5
Cl6—Cd1—Cl3	170.78 (3)	C5—C9—H9B	109.5
Cl2—Cd1—Cl3	92.88 (3)	H9A—C9—H9B	109.5
Cl4—Cd1—Cl3	89.14 (3)	C5—C9—H9C	109.5
Cl6—Cd1—Cl5^i^	103.43 (3)	H9A—C9—H9C	109.5
Cl2—Cd1—Cl5^i^	89.29 (3)	H9B—C9—H9C	109.5
Cl4—Cd1—Cl5^i^	87.06 (3)	C12—C11—C10	122.2 (3)
Cl3—Cd1—Cl5^i^	85.78 (3)	C12—C11—H11	118.9
Cl6—Cd1—Cl5	89.11 (3)	C10—C11—H11	118.9
Cl2—Cd1—Cl5	93.06 (3)	C1—C7—H7A	109.5
Cl4—Cd1—Cl5	91.00 (3)	C1—C7—H7B	109.5
Cl3—Cd1—Cl5	81.68 (3)	H7A—C7—H7B	109.5
Cl5^i^—Cd1—Cl5	167.335 (9)	C1—C7—H7C	109.5
Cd1^ii^—Cl5—Cd1	159.99 (4)	H7A—C7—H7C	109.5
C15—N2—H2A	109.5	H7B—C7—H7C	109.5
C15—N2—H2B	109.5	C1—C2—C3	122.4 (3)
H2A—N2—H2B	109.5	C1—C2—H2	118.8
C15—N2—H2C	109.5	C3—C2—H2	118.8
H2A—N2—H2C	109.5	C20—C21—C22	116.4 (4)
H2B—N2—H2C	109.5	C20—C21—C26	121.2 (3)
C14—C15—C10	123.0 (3)	C22—C21—C26	122.4 (3)
C14—C15—N2	118.4 (3)	C23—C22—C21	123.4 (3)
C10—C15—N2	118.6 (3)	C23—C22—N3	118.6 (3)
C22—N3—H3A	109.5	C21—C22—N3	118.0 (3)
C22—N3—H3B	109.5	C20—C19—C24	118.6 (4)
H3A—N3—H3B	109.5	C20—C19—C25	121.6 (4)
C22—N3—H3C	109.5	C24—C19—C25	119.8 (5)
H3A—N3—H3C	109.5	C13—C12—C11	118.7 (3)
H3B—N3—H3C	109.5	C13—C12—C18	120.3 (4)
C1—C6—C5	122.8 (3)	C11—C12—C18	121.0 (4)
C1—C6—N1	118.9 (3)	C12—C13—C14	121.4 (3)
C5—C6—N1	118.3 (3)	C12—C13—H13	119.3
C15—C10—C11	116.6 (3)	C14—C13—H13	119.3
C15—C10—C16	122.0 (3)	C19—C20—C21	122.7 (4)
C11—C10—C16	121.3 (3)	C19—C20—H20	118.7
C6—C5—C4	117.4 (3)	C21—C20—H20	118.7
C6—C5—C9	121.9 (3)	C21—C26—H26A	109.5
C4—C5—C9	120.6 (3)	C21—C26—H26B	109.5
C6—N1—H1A	109.5	H26A—C26—H26B	109.5
C6—N1—H1B	109.5	C21—C26—H26C	109.5
H1A—N1—H1B	109.5	H26A—C26—H26C	109.5
C6—N1—H1C	109.5	H26B—C26—H26C	109.5
H1A—N1—H1C	109.5	C14—C17—H17A	109.5
H1B—N1—H1C	109.5	C14—C17—H17B	109.5
C2—C1—C6	117.2 (3)	H17A—C17—H17B	109.5
C2—C1—C7	120.0 (3)	C14—C17—H17C	109.5
C6—C1—C7	122.8 (3)	H17A—C17—H17C	109.5
C10—C16—H16A	109.5	H17B—C17—H17C	109.5
C10—C16—H16B	109.5	C19—C24—C23	121.5 (4)
H16A—C16—H16B	109.5	C19—C24—H24	119.2
C10—C16—H16C	109.5	C23—C24—H24	119.2
H16A—C16—H16C	109.5	C3—C8—H8A	109.5
H16B—C16—H16C	109.5	C3—C8—H8B	109.5
C22—C23—C24	117.4 (4)	H8A—C8—H8B	109.5
C22—C23—C27	123.1 (3)	C3—C8—H8C	109.5
C24—C23—C27	119.6 (4)	H8A—C8—H8C	109.5
C23—C27—H27A	109.5	H8B—C8—H8C	109.5
C23—C27—H27B	109.5	C19—C25—H25A	109.5
H27A—C27—H27B	109.5	C19—C25—H25B	109.5
C23—C27—H27C	109.5	H25A—C25—H25B	109.5
H27A—C27—H27C	109.5	C19—C25—H25C	109.5
H27B—C27—H27C	109.5	H25A—C25—H25C	109.5
C3—C4—C5	122.1 (3)	H25B—C25—H25C	109.5
C3—C4—H4	118.9	C12—C18—H18A	109.5
C5—C4—H4	118.9	C12—C18—H18B	109.5
C4—C3—C2	118.1 (3)	H18A—C18—H18B	109.5
C4—C3—C8	121.9 (3)	C12—C18—H18C	109.5
C2—C3—C8	120.0 (3)	H18A—C18—H18C	109.5
C15—C14—C13	118.1 (3)	H18B—C18—H18C	109.5

Symmetry codes: (i) *x*−1/2, −*y*+1/2, −*z*+2; (ii) *x*+1/2, −*y*+1/2, −*z*+2.

### Hydrogen-bond geometry (Å, °)

**Table:**

*D*—H···*A*	*D*—H	H···*A*	*D*···*A*	*D*—H···*A*
N2—H2C···Cl3	0.89	2.26	3.129 (3)	166.
N3—H3B···Cl3	0.89	2.70	3.283 (3)	124.
N3—H3B···Cl5	0.89	2.62	3.158 (3)	119.
N2—H2A···Cl6^i^	0.89	2.40	3.250 (3)	160.
N3—H3A···Cl2^ii^	0.89	2.41	3.264 (3)	160.
N1—H1A···Cl4^iii^	0.89	2.43	3.278 (3)	160.
N1—H1B···Cl3^iv^	0.89	2.61	3.285 (3)	134.
N1—H1C···Cl2^iv^	0.89	2.43	3.306 (3)	169.

Symmetry codes: (i) *x*−1/2, −*y*+1/2, −*z*+2; (ii) *x*+1/2, −*y*+1/2, −*z*+2; (iii) *x*−1/2, −*y*+3/2, −*z*+1; (iv) *x*, *y*+1, *z*−1.
